# Circulating CitH3 Is a Reliable Diagnostic and Prognostic Biomarker of Septic Patients in Acute Pancreatitis

**DOI:** 10.3389/fimmu.2021.766391

**Published:** 2021-11-17

**Authors:** Baihong Pan, Yaozhen Li, Yu Liu, Wei Wang, Gengwen Huang, Yang Ouyang

**Affiliations:** Department of General Surgery, Xiangya Hospital, Central South University, Changsha, China

**Keywords:** acute pancreatitis, sepsis, citrullinated histone H3, peptidyl arginine deiminase, diagnosis, prognosis

## Abstract

**Purpose:**

Acute pancreatitis (AP) is an inflammatory disease. AP starts with sterile inflammation and is often complicated with critical local or systemic infection or sepsis in severe cases. Septic AP activates peptidyl arginine deiminase (PAD) and citrullinates histone H3 (CitH3), leading to neutrophil extracellular trap (NET) formation. Investigating the role of NETs and underlying mechanisms in septic AP may facilitate developing diagnostic and therapeutic approaches. In this study, we sought to identify the expression of CitH3 in septic AP patients and to analyze the correlation of CitH3 concentration with NET components as well as clinical outcomes.

**Methods:**

Seventy AP patients with or without sepsis (40 septic cases, 30 nonseptic cases) and 30 healthy volunteers were recruited in this study. Concentration of NET components (CitH3 and double-strain DNA) and key enzymes (PAD2/4) were measured. Clinical and laboratory characteristics of patients were recorded and analyzed.

**Results:**

Levels of CitH3 were elevated significantly in septic AP patients compared with those in nonseptic AP and healthy volunteers. The area under the curve (AUC, 95% confidence interval) for diagnosing septic AP was 0.93 (0.86–1.003), and the cutoff was 43.05 pg/ml. Among septic AP cases (*n* = 40), the concentration of CitH3 was significantly increased in those who did not survive or were admitted to the intensive care unit, when compared with that in those who survived or did not require intensive care unit. Association analysis revealed that CitH3 concentration was positively correlated with PAD2, PAD4, dsDNA concentration, and Sequential Organ Failure Assessment scores.

**Conclusion:**

CitH3 concentration increased in septic AP patients and was closely correlated with disease severity and clinical outcomes. CitH3 may potentially be a diagnostic and prognostic biomarker of septic AP.

## 1 Introduction

Acute pancreatitis (AP) is an inflammatory disease caused by digestive enzyme activation and self digestion (1). AP causes local or systemic sterile inflammation at early stages; however, up to 40%–70% of AP patients develop pancreatitis-related infection during the late stage or sepsis in severe cases (2). Sepsis is the leading cause of death in AP; thus, early diagnosis of septic AP and prompt initiation of treatments are the key to improving outcomes (3). Microbe culture is the gold standard to distinguish septic pancreatitis from sterile pancreatitis (4, 5), but the test is time consuming and can be unreliable because of false-positive and false-negative results. Identification of reliable circulating biomarkers to diagnose sepsis is therefore highly desirable. Procalcitonin (PCT) has been recognized as a promising sepsis biomarker and is widely used in the clinic (6). However, diagnostic efficacy of PCT is compromised significantly because of its nonspecificity (7). Therefore, novel strategies to identify key pathological signal pathways and improve the rapid diagnosis of septic AP are desperately needed.

Neutrophil extracellular traps (NETs) play a key role in the pathophysiology of septic AP. AP recruits and activates neutrophils, releasing nuclear and cytosolic components such as DNA, histones, and antimicrobial enzymes (8). This process is called NETosis (9). NETs combat infection in septic AP patients by trapping and killing invading microbes. However, recent studies revealed that NETs may also be involved in the pathogenesis of AP through inducing trypsin activation and promoting systemic inflammatory responses and tissue damage (10–13). Moritz et al. have reported in *Nature Communications* that NETs aggregate and occlude pancreatic ducts, driving pancreatitis. Excessive NETs exacerbate sepsis (14), while blockade of NETosis has been proven to ameliorate AP and improve outcomes (15–17).

Previous studies have proven that peptidylarginine deiminase (PAD) activation and downstream citrullination of histone H3 (CitH3) triggers NET formation (18, 19). Among PAD isoforms, PAD2/4 are mainly expressed in immune cells and are involved in the signaling pathway of infectious NETosis. Studies have shown that inhibition of PAD2 or PAD4 significantly decreases sepsis-induced NET formation (20, 21). PAD2/4 activation and CitH3 generation stimulate sepsis-specific gene expression and signaling transduction (22). We have proven that serum levels of CitH3 are increased significantly in both cecal ligation and puncture (CLP) and lipopolysaccharide (LPS)-induced septic mice but not in sterile inflammatory mice, and CitH3 may thus be a reliable diagnostic biomarker of sepsis (23, 24). Moreover, high levels of CitH3 in blood worsen sepsis, while clearance of CitH3 by anti-CitH3 antibodies can significantly improve survival of septic animals (25, 26) through attenuation of sepsis-induced acute lung injury and inflammatory cytokine cascade.

Investigating the pathogenic role of PADs and CitH3 in septic AP patients will facilitate the development of novel diagnostic biomarkers and therapeutic targets. In this study, we sought to measure the concentration of CitH3, to identify the correlation of CitH3 with PAD2/4, and to analyze the potential relationship between CitH3 levels and disease severity and outcomes.

## 2 Material and Methods

### 2.1 Enrollment of Study Objects

Forty septic AP patients (SP), 30 noninfectious AP patients (NIP), and 30 healthy volunteers (HV) who were admitted into Xiangya Hospital, Central South University were enrolled in this study. Patients in the SP group were adults who were diagnosed with AP (3) and met the consensus definition for sepsis: confirmed infection (positive microbe culture of peripheral blood or peripancreatic tissue); two or more systemic inflammatory response criteria; and organ dysfunction (27). NIP adult controls were diagnosed with AP for less than 72 h without any sign of infection (fever, radiological sign of peripancreas infection, and negative microbe culture). HV were ambulatory age- and sex-matched adults who had no chronic medical problems or any medications. All procedures were approved by the Institute Review Board of Xiangya Hospital, Central South University. Patients were informed, and consent forms were obtained for research purposes. Clinical data were collected at the time of confirmation of septic or noninfectious AP. Sixty-day survival rates were determined through follow-up study.

### 2.2 Blood Sample Analysis

Blood samples from the SP group were collected at the time when AP patients met the sepsis criteria. Blood samples from the NIP group were collected when AP was confirmed for less than 72 h without signs of infection. Samples were processed by trained researchers and were stored at −80°C until the time of assay.

Quantification of CitH3 was performed by an enzyme-linked immunosorbent assay (ELISA) that we developed previously (23). In brief, anti-CitH3 monoclonal antibody raised by CitH3 peptides (R2+R8+R17+R26, 30 amino acids) (25) was coated onto 96-well plates as capture antibody and was then blocked by protein-free blocking buffer (Thermo Scientific, Rockford, IL, USA). Blood samples or CitH3 peptide (R2+R8+R17+R26) were incubated in the wells for 2 h, followed by incubation with rabbit anti-CitH3 polyclonal antibody (Abcam, Cambridge, MA, USA). Next, 96-well plates were probed with antirabbit horseradish peroxidase (HRP)-conjugated IgG (Jackson Immuno-Research, West Grove, PA, USA). 3,3′,5,5′-Tetramethylbenzidine (TMB, Thermo Fisheer Scientific, Waltham, MA, USA) was added into wells and incubated for 20 min at room temperature in the dark before adding stop solution (R&D Systems Inc., Minneapolis, MN, USA).

PAD2 and PAD4 were measured using commercial ELISA kits (PAD2, #501450, Cayman Chemical, Ann Arbor, MI, USA; PAD4, #501460, Cayman Chemical). NET-associated double-stranded DNA (dsDNA) was quantified by a PicoGreen assay kit (Invitrogen, San Diego, CA, USA). All procedures were performed in accordance with the manufacturer’s instructions.

### 2.3 Clinical and Laboratory Data Collection

Clinical and laboratory data including age, sex, hospital stay, intensive care unit (ICU) stay, lactate, and PCT were obtained during hospitalization. Sequential organ failure assessment (SOFA) score and its components were recorded based on laboratory results at the same time as blood sample collection (27).

### 2.4 Statistical Analysis

Categorical values were presented as numbers (percentages), and continuous variables were presented as means (standard deviations) or medians (interquartile ranges). One-way analysis followed by Bonferroni’s multiple comparison test was performed for comparison between three or more groups. The Mann-Whitney *U* test was performed for comparisons between two groups. Receiver operating characteristic curves (ROC) were used to identify the diagnostic and prognostic efficacy of CitH3 and PCT. Optimal cutoff values were determined when the Youden index (sensitivity + specificity − 1) was maximized. Correlation between CitH3 and PAD2, PAD4, dsDNA, and SOFA scores were determined by Pearson’s regression model. Analyses were performed with GraphPad Prism 7 (GraphPad Software Inc., La Jolla, CA, USA). *p* < 0.05 was defined as statistically significant.

## 3 Results

### 3.1 Baseline Characteristics

A total of 100 individuals were enrolled in this study, including 40 septic AP patients, 30 noninfectious AP patients and 30 healthy volunteers (SP, NIP, and HV groups, respectively) from Xiangya Hospital, Central South University. Baseline characteristics are presented in **Table 1** (**Supplementary Materials**).

**Table 1:** Baseline demographics and clinical characteristics for septic AP patients and noninfectious AP patients.

Variables	SP (*n* = 40)	NIP (*n* = 30)	HV (*n* = 30)	*p*-value
**Age (mean (SD))**	47.3 (12.37)	55.8 (10.76)	50.9	>0.05
**Female (*n* (%))**	8 (20)	8 (26.7)	9 (30)	>0.05
**PCT (ng/ml, median (IQR))**	0.43 (0.17–0.99)	0.27 (0.2–0.8)	0 (0–0.08)	>0.05
**Lactate (mmol/L, median (IQR))**	2 (1.8–2.4)	1.6 (1.3–1.9)		<0.05
**CitH3 (pg/ml, median (IQR))**	127.3 (82.4–210.2)	27.5 (21–33.5)	26.5 (20.5–39.75)	<0.05
**PAD2 (ng/ml, median (IQR))**	3.03 (2.1–3.68)	0.32 (0.21–0.49)	0.16 (0.1–0.26)	<0.05
**PAD4 (ng/ml, median (IQR))**	3.11 (2.53–4.71)	0.28 (0.14–0.51)	0.12 (0.07–0.21)	<0.05
**dsDNA (ng/ml, median (IQR))**	913.5 (812–1063)	257 (232.5–294)	89 (65–100)	<0.05
**Survivals (*n* (%))**	33 (82.5)	30 (100)		<0.05
**Length of hospital stay (day, median (IQR))**	28.5 (20.8–57)	13.5 (11.3–16.8)		<0.05
**Length of ICU stay (day, median (IQR))**	4 (1–7.25)	0 (0–0)		<0.05
**Total SOFA score (median (IQR))**	5 (3–8)	1 (1–2)		<0.05
**Respiratory SOFA (median (IQR))**	2 (2–3)	1 (1–2)		<0.05
**Renal SOFA (median (IQR))**	1 (0–3)	0 (0–0)		<0.05
**Cardiovascular SOFA (median (IQR))**	0 (0–0)	0 (0–0)		<0.05
**Neurologic SOFA (median (IQR))**	0 (0–0)	0 (0–0)		<0.05
**Hepatic SOFA (median (IQR))**	1 (0–1)	0 (0–0)		<0.05
**Coagulation SOFA (median (IQR))**	0 (0–1)	0 (0–0)		<0.05

Categorical variables are shown as number (%). For continuous variables, normally distributed values are shown as mean (SD) while not normally distributed are shown as median (interquartile range Q1/Q3). AP, acute pancreatitis; PCT, procalcitonin; CitH3, citrullinated histone H3; PAD2/4, peptidylarginine deiminase 2/4; ICU, intensive care unit; SOFA, sequential organ failure assessment.

No significant difference in age and sex were observed between SP, NIP, and HV groups. However, patients in the SP group experienced increased levels of lactate, prolonged hospital and ICU stay, and decreased survival rate. Furthermore, patients in the SP group experienced higher SOFA score (total, respiratory, renal, cardiovascular, neurologic, hepatic, and coagulation SOFA), which suggested that the SP group suffered more severe multiple organ failure.

### 3.2 Serum CitH3 as a Diagnostic Biomarker of Septic AP Patients

**Figure 1:**
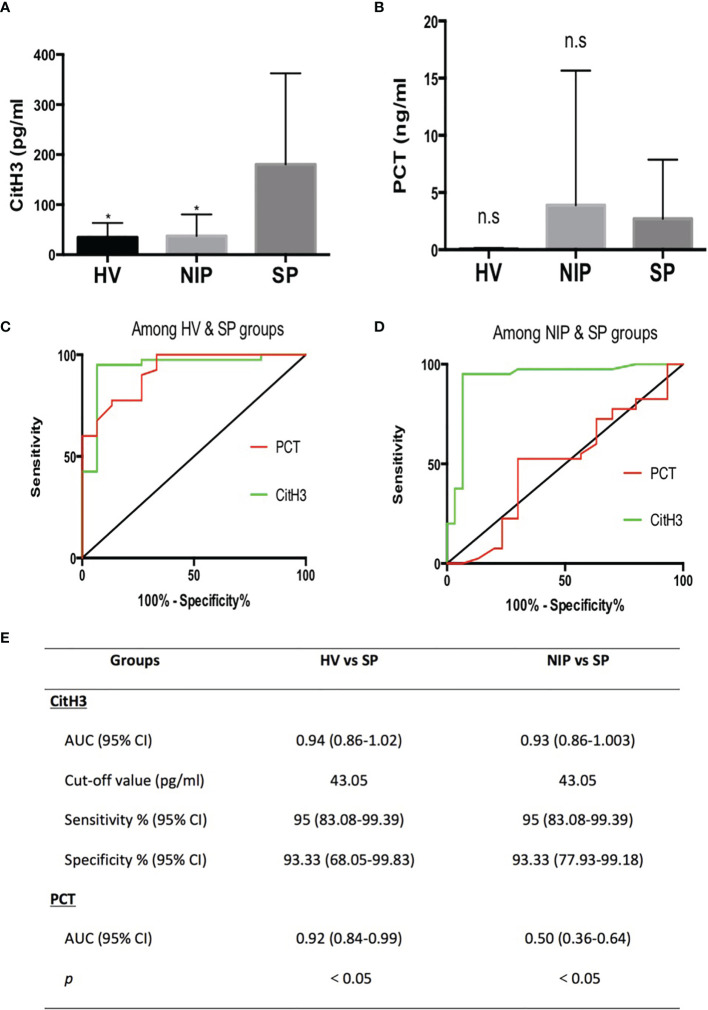
All CitH3 concentration is elevated in septic AP patients and may be a reliable diagnostic biomarker of sepsis in AP. Blood samples from SP, NIP, and HV groups were collected. CitH3 and PCT concentration were measured by ELISA kit. CitH3 concentration was increased significantly in the SP group compared with that in the HV and NIP groups **(A)**. No significant differences in PCT concentration were identified among SP, NIP, and HV groups **(B)**. Receiver operating characteristic curves analysis of CitH3 and PCT to diagnose septic AP compared with that of the HV **(C)** and NIP group **(D)**. Detailed information of receiver operating characteristic curve analysis is presented in the table **(E)**. ^*^ *p* < 0.05; ns, non-significant.

As mentioned, severe AP patients often are complicated with local or systemic infection at late stages. On set of sepsis usually indicates poor outcome. Thus, early diagnosis of sepsis in AP patients is of significant value. In this study, levels of CitH3 were dramatically increased in SP patients compared with both HV and NIP groups (**Figure 1A**) [median (interquartile range): SP 127.3 pg/ml (82.4–210.2), NIP 27.5 pg/ml (21–33.5), HV 26 pg/ml (22.5–35.5); *p* < 0.0001 for SP vs. NIP, *p* = 0.001 for SP vs. HV]. No significant difference was observed between HV and NIP groups. This suggests that CitH3 may distinguish septic AP cases from nonseptic AP cases.

Clinically, PCT is a widely used biomarker for diagnosing infection. Comparison study of PCT and CitH3 were conducted to evaluate the diagnostic value of CitH3. As shown in **Figure 1B**, no significant change in PCT concentration was observed among HV, NIP, and SP groups. Furthermore, ROC curve analysis was conducted in both PCT and CitH3. When comparing HV and SP groups (**Figure 1C**), the areas under the curves (AUCs) and 95% confidence interval (CI) for CitH3 and PCT were 0.94 (0.86–1.02) and 0.92 (0.84–0.99), respectively (*p* < 0.0001). When comparing NIP and SP groups (**Figure 1D**), AUCs (95% CI) for CitH3 and PCT were 0.93 (0.86–1.003) and 0.50 (0.36–0.64), respectively (*p* < 0.0001). This suggests that CitH3 exerts better diagnostic power compared with PCT, especially in distinguishing septic AP patients from noninfectious AP suffers.

Based on the Youden index, CitH3 concentration above 43.05 pg/ml was suggestive of sepsis in AP patients compared with that in HV and NIP patients (**Figure 1E**).

### 3.3 Serum CitH3 as a Prognostic Biomarker of Septic AP Patients

Survival rate, admittance to the ICU, and SOFA score are the hallmarks of prognosis. All SP AP patients were subgrouped into survival (*n* = 33) and death (*n* = 7) groups or non-ICU (*n* = 31) and ICU (*n* = 9) groups.

#### 3.3.1 Survival Group vs. Death Group

**Figure 2:**
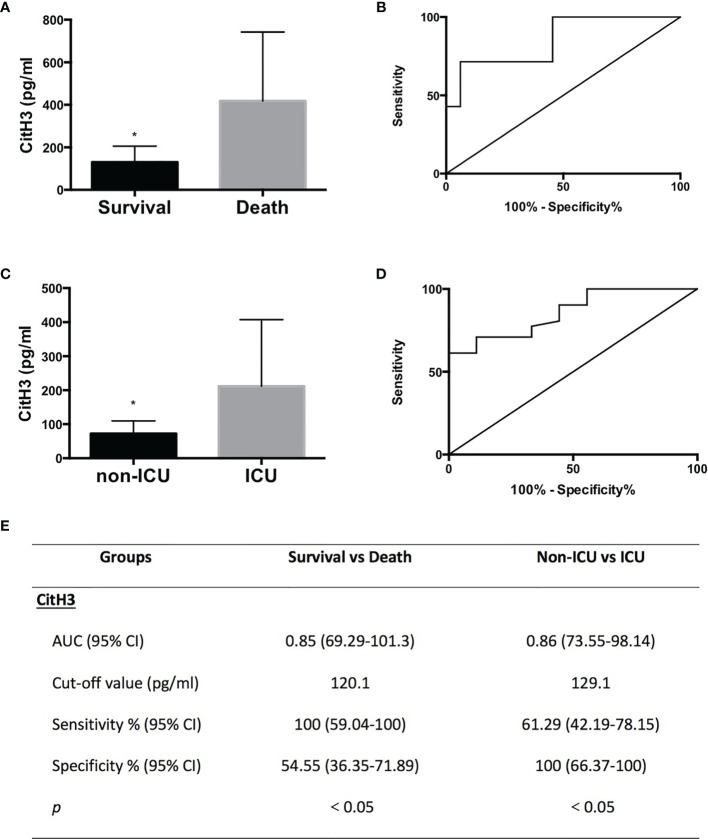
All CitH3 may be a promising prognostic biomarker in septic AP patients. AP patients were subgrouped into survival and death groups **(A)** or ICU (cases admitted into ICU) and non-ICU (cases not admitted into ICU) groups **(C)**. Concentration of CitH3 was measured by ELISA. Levels of CitH3 were increased significantly in the death group and ICU group compared with those in survival group and non-ICU group, respectively. Receiver operating characteristic curve analysis of CitH3 to distinguish survival and death cases **(B)** and cases admitted or not into ICU **(D)**. The detailed information of receiver operating characteristic curve analysis is presented in the table **(E)**. ^*^ *p* < 0.05.

Levels of CitH3 were also increased dramatically in dead cases compared with those in the survival group (**Figure 2A**) (*p* = 0.0024). ROC curve analysis revealed that the AUC (95% CI) was 0.85 (69.29–101.3) when comparing survival and death groups (**Figure 2B**). The cutoff value was 120.1 pg/ml, which suggested that SP patients with CitH3 above 120.1 pg/ml may experience poorer outcome than those with CitH3 lower than 120.1 pg/ml (**Figure 2E**).

#### 3.3.2 Non-ICU Group vs. ICU Group

Levels of CitH3 were also increased dramatically in cases who were admitted to the ICU compared with those in cases who did not require intensive care (**Figure 2C**) (*p* = 0.0006) (**Figure 2E**). ROC curve analysis showed that the AUC (95% CI) was 0.86 (73.55–98.14) when comparing non-ICU and ICU groups (**Figure 2D**). The cutoff value was 129.1 pg/ml, suggesting that septic AP patients with CitH3 above 129.1 pg/ml may suffer a higher possibility of being admitted to the ICU than those with CitH3 lower than 129.1 pg/ml (**Figure 2E**).

#### 3.3.3 SOFA Score

**Figure 3:**
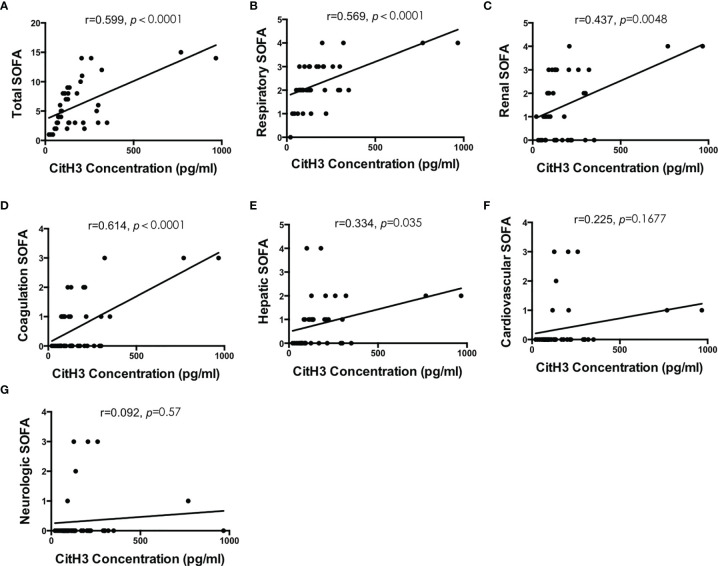
Blood concentration of CitH3 is positively correlated with SOFA score. Association between CitH3 concentration and total SOFA **(A)**, respiratory SOFA **(B)**, renal SOFA **(C)**, coagulation SOFA **(D)**, hepatic SOFA **(E)**, cardiovascular SOFA **(F)**, and neurologic SOFA **(G)** score was determined by *Pearson’s regression* analysis.

Total SOFA score comprises respiratory, cardiovascular, renal, coagulation, hepatic, and neurologic scores and is a widely used tool for quick assessment of organ dysfunction (28). The higher the score, the more severe the organ dysfunction. Positive correlations were found between CitH3 concentration and total SOFA score (*r* = 0.599, *p* < 0.0001), respiratory SOFA score (*r* = 0.569, *p* < 0.0001), renal SOFA score (*r* = 0.437, *p* = 0.0048), coagulation SOFA score (*r* = 0.614, *p* < 0.0001), and hepatic SOFA score (*r* = 0.334, *p* = 0.035) (**Figures 3A**–**E**). No obvious correlation was observed between CitH3 concentration and cardiovascular SOFA score (*r* = 0.225, *p* = 0.1677), and neurologic SOFA score (*r* = 0.092, *p* = 0.57) (**Figures 3F, G**).

### 3.4 Correlation of Serum CitH3 With NETosis Pathway Components

**Figure 4:**
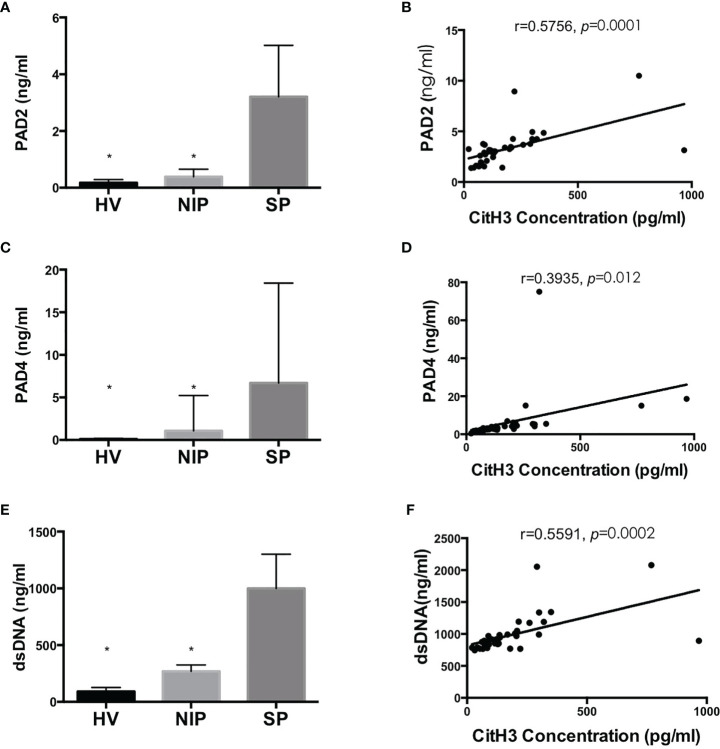
Blood concentration of PAD2/4 and dsDNA is increased significantly in SP group and is positively correlated with CitH3 concentration. Levels of PAD2 **(A)**, PAD4 **(C)**, and dsDNA **(E)** were using commercialized kits. Association between CitH3 concentration and PAD2 **(B)**, PAD4 **(D)**, and dsDNA **(F)** were determined by *Pearson’s regression* analysis *p < 0.05.

As mentioned above, CitH3 mainly originates from NETosis. PAD2/4 activation followed by CitH3 and dsDNA complex release is the key mechanism. As shown in **Figures 4A, C, E**, concentration of PAD2, PAD4, and dsDNA were significantly increased in the SP group compared with those in the NIP group and HV groups (*p* < 0.05). Correlation analysis revealed that that serum CitH3 concentration was positively correlated with PAD2, PAD4, and dsDNA [(*r* = 0.5756, *p* = 0.0001), (*r* = 0.3935, *p* = 0.012), (*r* = 0.5591, *p* = 0.0002), respectively] (**Figures 4B, D, E**). These data strongly suggest that elevated serum CitH3 may be released by netting neutrophils in septic AP patients.

## 4 Discussion

Sepsis is a severe complication in the late stage of AP and accounts for majority of fatal AP cases. Thus, early diagnosis of septic AP and prompt initiation of treatments are essential for improving prognosis. We have previously shown that serum CitH3 is a reliable diagnostic biomarker for sepsis in a murine model (23), and clearance of CitH3 by enzymatic inhibition or antibody neutralization improves survival in septic mice (21, 25). The goal of the current study was to identify whether the diagnostic efficacy of CitH3 for sepsis was conserved in humans. Furthermore, we planned to determine whether the elevated CitH3 concentration in humans is originated from NETosis, because this has been confirmed many times in rodent models. In this study, we found that (1) circulating CitH3 was increased dramatically in septic AP but not in sterile AP or healthy subjects; (2) concentration of CitH3 was positively correlated with PAD2/PAD4 expression and serum dsDNA concentration; (3) serum levels of CitH3 correlated positively with disease severity and clinical outcomes. Therefore, CitH3 may represent a diagnostic and prognostic biomarker of septic AP, mediating the pathogenesis of sepsis in AP.

Neutrophils are the most abundant leukocytes and have been confirmed as the frontline of host immune defense. Classical mechanisms of killing invasive microbes include phagocytosis, degranulation, and reactive oxygen species. Recently, however, a novel antimicrobe strategy—NETosis—was discovered by Brinkmann et al. (9). When stimulated by invading microbes, neutrophils release NETs, a net-like structure consisting of DNA, histones (particularly CitH3), and granule proteins inside neutrophils. NET can trap pathogens by histones and various antipathogen proteinases. Excessive NET formation, however, has been shown to be related to tissue damage, organ dysfunction, and poor outcome in different diseases. Studies have suggested that NETs mediate pathogenesis of AP, and clearance of NETs alleviates pancreatic injury and exerts protective effects (8, 12). Our data further supported these findings. We found that NET components such as CitH3 and dsDNA were increased significantly in septic AP and were closely correlated with disease severity. Furthermore, the positive association between CitH3 and PAD2/4 revealed that elevated CitH3 may be caused by NETosis in septic AP patients.

NETosis is generally associated with activation of PADs and histone citrullination. It has been well documented that only PAD2 and PAD4 isoforms are expressed in immune cells and can translocate into the nucleus to citrullinate histones, while the crucial role of PAD4 in NETosis has been illustrated by plenty of researchers (18, 29, 30). However, whether PAD2 can promote NETosis remains unclear. Recently, Li et al. (31, 32) published two papers suggesting that PAD2 also participates in NETosis and PAD2 blockade significantly improves survival of septic mice. In the present study, we also found that increased levels of CitH3 positively correlated with PAD2 concentration (*r* = 0.5756). Furthermore, concentration of CitH3 positively correlates with the severity of septic AP. Collectively, we believe that PAD2/4 activation leads to excessive CitH3/NETs release in septic AP patients, thus deteriorating the outcomes.

The most crucial characteristic of a diagnostic biomarker is specificity. As previously described, nonspecificity to sepsis is the major weakness of PCT (7). In this study, we also found that PCT was not specific to sepsis. The concentration of PCT increased not only in the SP group but also in the NIP group without a statistically significant difference (**Figure 1B**). Based on our data, CitH3 may be a more reliable diagnostic biomarker of sepsis compared with PCT. Lactate is widely measured in clinical situations and is a helpful indicator of disease severity and outcome. However, lactate is a response to various acute diseases (myocardial infarction, trauma, shock, etc.) and fails to be specific to sepsis. As shown in **Table 1**, patients in both SP and NIP groups experienced increased lactate concentrations.

This study has several limitations. For most cases, concentration of CitH3 was only identified at the time of diagnosis. Periodical measurement of CitH3 levels may be more informative of clinical course and responses to treatments. Tracking of CitH3 changes during hospitalization may more accurately reflect the diagnostic efficacy of CitH3. Additionally, this study only showed clinical data and no animal models were involved. More detailed experiments using animal models to illustrate the presence of NETs may be more convincing. Future studies should be performed to determine whether PAD2/4-CitH3 pathway could be a therapeutic target for septic AP.

In conclusion, this clinical study demonstrates that serum CitH3 increases in septic but not in sterile AP patients. Increased levels of CitH3 are closely correlated with disease severity and clinical outcomes. Association study of CitH3 and PAD2/4 further revealed that circulating CitH3 may originate from septic AP-induced NETs. Overall, serum CitH3 may be a reliable diagnostic and prognostic biomarker of septic AP. Understanding the mechanistic role of the PAD-CitH3 pathway in the pathogenesis of septic AP may facilitate the development of novel diagnostic and therapeutic approaches.

## Data Availability Statement

The original contributions presented in the study are included in the article/**Supplementary Material**. Further inquiries can be directed to the corresponding authors.

## Ethics Statement

The studies involving human participants were reviewed and approved by the Institute Review Board of Xiangya Hospital, Central South University. The patients/participants provided their written informed consent to participate in this study.

## Author Contributions

BP, GH, and YO designed the study. BP, YZL, and YL performed the experiments and interpreted the data. BP wrote the manuscript, which was critically revised by WW and YO. BP and YO acquired the funding. All authors contributed to the article and approved the submitted version.

## Funding

This work was funded by grants from Young Research Funding of Xiangya Hospital, Central South University (2019Q10) and the National and Science Foundation of Hunan Province (2020JJ4902).

## Conflict of Interest

The authors declare that the research was conducted in the absence of any commercial or financial relationships that could be construed as a potential conflict of interest.

## Publisher’s Note

All claims expressed in this article are solely those of the authors and do not necessarily represent those of their affiliated organizations, or those of the publisher, the editors and the reviewers. Any product that may be evaluated in this article, or claim that may be made by its manufacturer, is not guaranteed or endorsed by the publisher.
